# Can aged *Camellia oleifera* Abel oil truly be used to treat atopic dermatitis?

**DOI:** 10.3389/fphar.2024.1449994

**Published:** 2024-12-04

**Authors:** Xi-Lin Ouyang, Zhang-Lin Yuan, Xiao-Bing Chen, Hong-Wan Gan, Sen-Hui Guo, Juan Cai, Jing-Jing Zhong

**Affiliations:** ^1^ Department of Pharmacy, Gannan Healthcare Vocational College, Ganzhou, China; ^2^ Department of Dermatology, Ganzhou People’s Hospital, Ganzhou, China

**Keywords:** *Camellia oleifera* Abel oil, GC/Q-TOF MS, atopic dermatitis, fatty acids, molecular docking, major metabolites

## Abstract

Atopic dermatitis is an inflammatory skin condition characterized by erythema, eruption, lichenification, and pruritus. Aged *Camellia oleifera* Abel oil, an effective empirical plant oil utilized by the Gannan Hakka people in China to alleviate the symptoms of atopic dermatitis. However, no scientific studies have been reported to prove whether this oil is truly effective. We conducted this study to confirm whether aged *C. oleifera* oil could alleviate the symptoms of 2,4-dinitrochlorobenzene (DNCB)-induced atopic dermatitis in mice. Differences in the thickness and weight of the right and left ears were measured. ELISA was used to determine the serum levels of the inflammatory factors IL-4, IgE, IFN-γ, and TNF-α. HE staining was performed to observe inflammatory cell infiltration in the mouse skin lesions. In addition, the metabolites of aged *C. oleifera* oils were analyzed, and molecular docking was used to assess the binding affinity of the major metabolites to filaggrin, a protein involved in skin barrier function. Animal studies showed that aged *C. oleifera* oil significantly improved the symptoms of atopic dermatitis. HE staining and measurement of inflammatory factor levels revealed similar results. A total of 41 metabolites were tentatively identified in the oil, with fatty acids emerging as the major metabolites. Molecular docking confirmed that the three most abundant fatty acids, i.e., oleic acid, *n*-hexadecanoic acid, and octadecanoic acid, bind well to filaggrin. Our results suggest that aged *C. oleifera* oils can be used to ameliorate the symptoms of atopic dermatitis. Fatty acids may be the major active metabolites responsible for the observed therapeutic effects by reducing transdermal water loss, increasing skin hydration, alleviating DNCB-induced skin barrier alterations, and eliminating itchy scratching caused by dry skin.

## 1 Introduction

Atopic dermatitis (AD), also known as eczema, is a common skin condition characterized by reddening, swelling, itching, blistering, and erosion of the skin (Clebak et al., 2023). The intense itching associated with AD can disrupt sleep, and scratching often exacerbates the condition, leading to infection and inflammation, which increases the difficulty of treatment (Garmhausen et al., 2013). There are various direct causes of AD, including genetic and environmental factors, skin barrier dysfunction, microbiological imbalances, immune dysregulation, skin inflammation, and environmental interactions; as a result, the prevalence of AD is increasing worldwide (Suaini et al., 2021). In addition to avoiding exposure to allergens, people with AD had to be treated with topical medications. These topical medications include topical steroid creams, antihistamines, topical immunomodulators, and ultraviolet light. However, long-term or excessive use of medications can cause obvious side effects such as skin atrophy, hyperpigmentation, abnormal hair growth, skin dryness, hormone dependency, and suppression of immune function (Leung et al., 2004). Therefore, it is important to treat AD not only to ensure that the drugs are safe and effective, but also to minimize side effects. It is worthwhile searching for anti-AD drugs from natural medicinal plants with low toxicity and significant efficacy.

People in many regions have traditionally used plant oils to treat AD (Linnamaa et al., 2010). Several natural plant oils, such as coconut oil, lavender oil, grapeseed oil, olive oil, and sunflower oil, have long been used as natural remedies for the treatment of various topical skin diseases (Foster et al., 2010; Simon et al., 2014; El-Salamouni et al., 2020). *Camellia oleifera* Abel oil (COO), an edible oil widely used in China, is also known as “Oriental olive oil”. The “Compendium of Materia Medica” (本草纲目, compiled by Shizhen Li, a pharmacologist in the Ming Dynasty), a pharmacological work that mainly introduces the basic theories of materia medica and traditional Chinese medicine, states that “COO is cool in nature, cools the blood, stops bleeding, clears heat, and removes toxins”. When fresh COO is stored for a long time, it becomes aged COO. It is generally believed by the Gannan Hakka people of China that the edible value of aged COO is decreased compared to fresh COO, while the medicinal effect is enhanced. The longer COO is stored, the better its medicinal effect. Modern pharmacological studies have shown that the active metabolites of COO that confer its anti-inflammatory properties may be total saponins (Zhang F. et al., 2022), and polyphenolic extracts have also been proposed to confer these properties (Chang et al., 2020). Unfortunately, polyphenolic compounds that are purified from active extracts have been shown to have low anti-inflammatory activity (Zhang et al., 2021).

In addition to the metabolites mentioned above, COO is rich in saturated and unsaturated fatty acids, including oleic acid, palmitic acid, palmitoleic acid, stearic acid, linoleic acid, linolenic acid, and eicosenoic acid (Yang et al., 2016; Zhang F. et al., 2022; Gao et al., 2024). Fatty acids are also the major metabolites of plant oils and may contribute to promoting wound healing and protecting skin barrier homeostasis through antioxidant and anti-inflammatory mechanisms (Lin et al., 2018). Therefore, to investigate whether aged COO can be used in the treatment of AD and whether fatty acids are the active metabolites with anti-AD activity, we established an animal model of AD to validate the therapeutic effects, determined metabolites of aged COO by GC/Q–TOF MS, and evaluated the possibility that the major metabolites exert anti-AD effects.

## 2 Materials and methods

### 2.1 Instruments and reagents

Cellular inflammatory factor levels were determined using a fully automatic microplate absorbance reader (WD-2102B, Beijing Liuyi). Compositional analysis was performed with an Agilent 7250 GC/Q–TOF MS system.

2,4-dinitrochlorobenzene (DNCB) was purchased from Sigma–Aldrich, St. Louis, MO, United States of America, and prednisone acetate (PA) was obtained from Shanghai Macklin Biochemical Co., Ltd. Shanghai, China. A mouse interferon gamma (IFN-γ) assay kit, mouse tumor necrosis factor alpha (TNF-α) assay kit, mouse immunoglobulin E (IgE) assay kit, and mouse interleukin 4 (IL-4) assay kit were purchased from Wuhan Elabscience Biotechnology Co., Ltd. Xylene, anhydrous ethanol, and 95% ethanol were purchased from Xilong Scientific Co., Ltd., China. Hematoxylin staining solution was purchased from Zhongshui Jinqiao Co., Ltd., China. Eosin staining solution was purchased from Solarbio Co., Ltd., Beijing, China. Hematoxylin was obtained from Servicebio (G1040 Servicebio, Wuhan, China).

### 2.2 Samples

The seeds of *C. oleifera* were collected from Xingguo County, Ganzhou, China, in November 2020. The flowchart of the seeds processed and extracted was as follows: *C. oleifera* seeds → Removing impurities → Sun drying → Hulling and separating → Further drying → Crushing → Steaming → Machine pressing → Filtering → *C. oleifera* oil. The COO was stored in a sealed container at room temperature.

### 2.3 Animals

Twenty 6-week-old male BALB/c mice were purchased from Spearfish (Beijing) Biotechnology Co. Ltd., license No. SCXK (Beijing) 2019–0010. The feeding conditions were as follows: temperature, 20°C–26°C; humidity, 40%–70%; and free access to drinking water. The mice were allowed to acclimate and were fed for 7 days, and then the mice were randomly divided into four groups (n = 5/group): a vehicle (acetone:olive oil = 34:1)-treated control group, a DNCB-treated group, a PA/DNCB-treated group, and a COO/DNCB-treated group. All the animal studies were conducted in accordance with a protocol that was approved by the Animal Ethics Committee of Gannan Healthcare Vocational College (approval number: 20220106).

### 2.4 Establishment of the AD model and treatment

**FIGURE 1:**
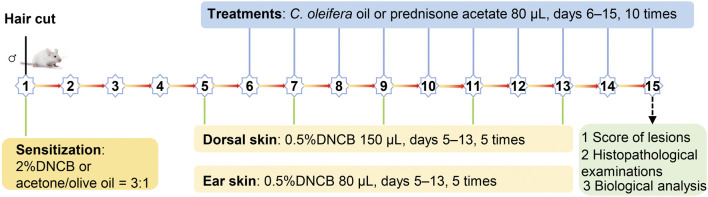
DNCB-induced AD model experimental design and treatment schedule.

The AD model was established as described in the literature (Kim et al., 2021). Briefly, the hair was removed from a 2-cm × 2-cm area on the back of BALB/c mice using electric clippers, and then the skin was sensitized with DNCB solution. In all groups except for the vehicle-treated control group, the skin on the back of mice was sensitized with 150 μL of 2% DNCB acetone-olive oil solution, while 10 μL of 2% DNCB acetone-olive oil solution was applied to the right ear on day 1. Subsequently, the mice in the COO/DNCB-treated group were stimulated with an acetone-olive oil mixture containing 0.5% DNCB on days 5, 7, 9, 11, and 13, while these mice were treated with corresponding amounts of aged COO on the skin of the backs and right ears from day 5 to day 14. As a control, the mice in the DNCB-treated group were treated with corresponding volumes of acetone-olive oil solution on the skin of the back and right ear, and the PA/DNCB-treated group was treated with prednisone acetate (Figure 1).

### 2.5 Measurement of indicators

#### 2.5.1 Observation of skin tissue appearance

The severity of erythema/hemorrhage, edema, abrasion/erosion, and dryness/scarring/inflammation on the back skin of mice in each group was observed and scored after the last treatment on day 15. The scoring criteria were as follows: 0, none; 1, mild; 2, moderate; 3, severe; and 4, very severe, with scores ranging from 0 to four for desquamation (dryness), edema (papules), erythema (hemorrhage), and epidermal peeling (scratches) (Wang et al., 2021).

#### 2.5.2 Weight and thickness differences between left and right ear

Animals were killed 24 h after the last treatment, and serum was collected for subsequent measurements of the IL-4, TNF-α, IgE, and TFN-γ levels. The mouse ears were carefully cleaned, and round ear slices were collected from the same position in the middle of the left and right ears using an 8-mm diameter metal punch. Each tissue sample was weighed using an electronic balance to calculate the difference in weight between the left and right ears, and the right ear sample was embedded in paraffin for the HE staining. In each group, the center thickness of the right and left ears was measured with calipers at the end of drug treatment, and the difference in thickness between the right and left ears was calculated.

#### 2.5.3 HE staining

Inflammatory cell infiltration into the skin lesions of mice was observed by HE staining to evaluate whether aged COOs can be used to treat AD. Right ear tissue from the mice was embedded in paraffin and sectioned at a thickness of 5 μm. The sections were incubated and dried in an oven at a constant temperature of 60°C for 2–4 h. After deparaffinization, the tissue sections were immersed in gradient ethanol solutions (75%, 85%, 95%Ⅰ, 95%Ⅱ, 100%Ⅰ, and 100%Ⅱ) for 60 min each for dehydration and hydrated in distilled water for a few minutes. Finally, the sections were stained with hematoxylin and eosin for 3–5 min each. After dehydration, the tissue sections were sealed with xylene, and inflammatory cell infiltration was observed under a microscope.

#### 2.5.4 Measurement of the serum IL-4, TNF-α, IgE, and TFN-γ concentrations

The levels of the inflammatory factors IL-4, IgE, IFN-γ, and TNF-α in the serum of mice were determined by ELISA. Whole-blood samples were collected in serum separator tubes and incubated at room temperature for 2 h. The samples were then centrifuged at 1000 *g* for 20 min. The serum was removed and transferred to microcentrifuge tubes and stored at 4°C in a refrigerator. IL-4, TNF-α, IgE, and TFN-γ levels in the mouse serum samples were measured as described in the ELISA kit instructions.

### 2.6 Metabolite analysis

#### 2.6.1 Pre-treatment of samples

Metabolite analysis of aged COO was performed according to the ConPhyMP guidelines (Heinrich et al., 2022). One hundred milligrams of COO sample were added to a 15-mL centrifuge tube, *n*-hexane was added to a final volume of 10 mL, and the mixture was vortexed for 1 min. Then, 1 mL of sample was transferred to a 1.5-mL centrifuge tube and centrifuged at 10,000 rpm for 5 min, after which the supernatant was transferred to an injection vial for measurement; *n*-hexane was used as the blank.

#### 2.6.2 Chromatographic conditions

The metabolites of the COO were analyzed via GC/Q–TOF MS. The chromatographic procedure involved an HP-5 MS UI column (30 m × 0.25 mm × 0.25 μm) with a shunt injection of carrier gas (He), an injection volume of 1.0 μL, and a column flow rate of 1 mL·min^−1^. The starting temperature was 40°C, and the temperature was increased from 40°C to 310°C at a rate of 10°C·min^−1^. The mass spectrometry conditions were as follows: full-scan mode, ionization source EI, ionization energy of 70 eV, transmission line temperature of 310°C, ion source temperature of 200°C, quadrupole temperature of 150°C, solvent delay time of 4 min, and scanning mass range of 20–550 amu.

### 2.7 Molecular docking

Three major fatty acids, namely, *n*-hexadecanoic acid, oleic acid, and octadecanoic acid, were used as small molecules for docking studies with filaggrin of the stratum corneum, which is closely related to AD (Earlia et al., 2019). In this docking study, the crystal structure files of filaggrin were obtained from the PDB database (www.rcsb.org), saved in pdb format, and then preprocessed as follows: removal of water of crystallization and non-standard peptide chains, merging of nonpolar hydrogens, merging of lone pairs of electrons, removal of solvent molecules, addition of all hydrogen atoms, recalculation of Gasteiger charge, and modification to Autodock four atom types. Then, the files were converted to pdbqt format using Autodock Tools 4.2.6 for backup (Morris et al., 2009). Using the CAS number of the small molecule of the ligand, the 3D structure file (.sdf format) of the ligand was obtained from the PubChem database (https://pubchem.ncbi.nlm.nih.gov/). The small molecules in the PubChem database have been optimized in terms of their preliminary structure and are in the ideal initial conformation for docking. The ligands were correctly protonated at pH = 7.4 (reference human pH: 7.35–7.45) and then converted to pdbqt format using OpenBabel 3.1.1 (O'Boyle et al., 2011). Docking was performed using the latest version of AutoDock vina 1.2.3 with the following parameters: algorithm exhaustiveness of 16, candidate docking results (num_modes) of 9, and energy threshold (energy_range) of ±3 kcal/mol.

### 2.8 Statistical analysis

All the experimental data were statistically analyzed using GraphPad Prism nine software. The results are expressed as the mean ± standard deviation (SD). Analysis of variance (ANOVA) was used to determine the statistical significance of differences between groups, with *p* < 0.05 considered to indicate statistical significance and *p* < 0.01 considered to indicate high statistical significance.

## 3 Results

### 3.1 Evaluation of the use of COO to improve skin appearance in AD model mice

**FIGURE 2:**
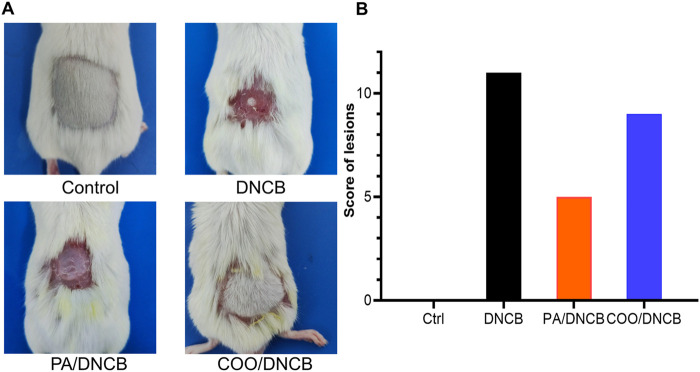
Typical appearance of mice in each group. **(A)** Effect of aged COO on the severity of DNCB-induced AD-like skin. Representative images of the vehicle-treated control group, DNCB-treated group, PA/DNCB-treated group, and COO/DNCB-treated group. **(B)** Scores of each group.

To investigate whether COO exerts anti-AD effects, we successfully established an AD model using a previously described method (Kim et al., 2021). DNCB-stimulated mice developed hemorrhages, edema, vesicles, scaling, and dryness, which caused constant itching, while the skin of all mice in the vehicle-treated control group began to regrow new hair. After the COO and PA were applied, the mice exhibited a similar skin appearance: the skin of the mice in the two groups improved in terms of hemorrhage and vesiculation, and the hair of some mice in the COO/DNCB-treated group began to regrow (Figure 2A). The PA/DNCB-treated group scored the highest, and the COO/DNCB-treated group had the second highest score, while the DNCB-treated group was the lowest. This result confirmed the ameliorative effect of COO on ameliorating the development of AD mice symptoms, but not as effective as the PA/DNCB-treated group (Figure 2B).

### 3.2 Effects of COO on the thickness and weight of the right and left ears of AD model mice

**FIGURE 3:**
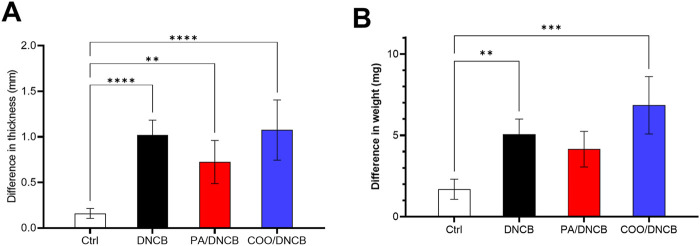
Differences in thickness and weight on the left and right ears of mice. **(A)** Difference in thickness between left and right ears **(B)** Difference in weight between left and right ears.

To determine the degree of skin swelling in AD model mice, differences in thickness of the right and left ears of the same mice were analyzed. Compared with those of mice in the vehicle-treated control group, the right ears of mice in other groups were swollen, and the differences in thickness of the left and right ears in each group were significant. Compared with those in the DNCB-treated group, the differences in the PA/DNCB-treated and COO/DNCB-treated groups were not significant. The thickness difference between the right and left ears of mice in the COO/DNCB-treated group was greater after the use of tea oil. The results showed that the right ear swelling did not seem to improve in the COO/DNCB-treated group of mice, and the severity of AD appeared to increase (Figure 3A).

Similar results were observed for differences in the weight of the right and left ears. The difference in skin weight between the right and left ears was greatest in mice treated with COO, but the difference was not statistically significant compared to mice in other groups. Moreover, the difference between the right and left ear weights of mice in all groups was not statistically significant (Figure 3B).

### 3.3 HE staining analysis of right ear tissue

**FIGURE 4:**
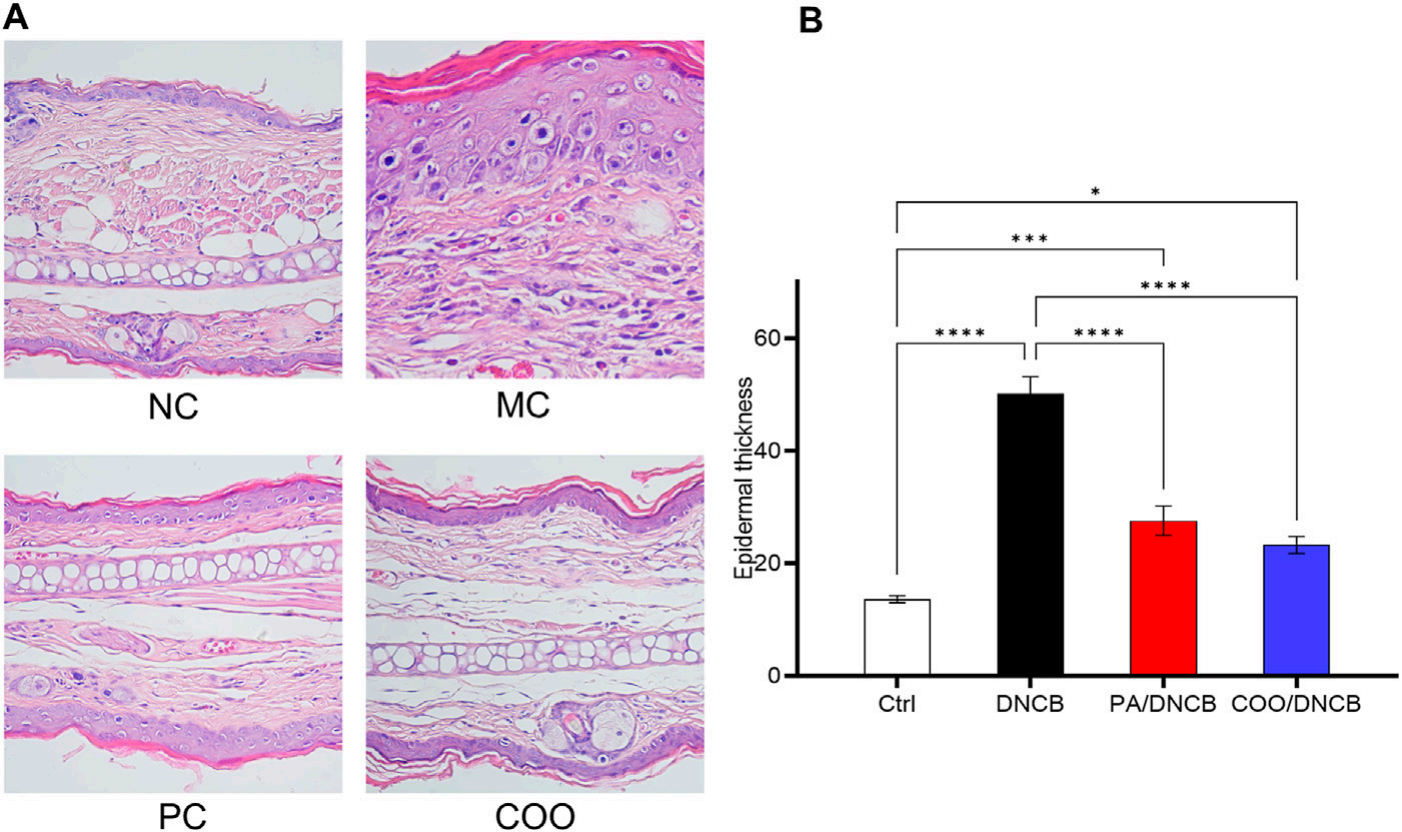
Histopathology of mouse ear skin after treatment with aged COO. **(A)** Results of HE staining on mouse skin tissues. **(B)** Comparison of epidermal layer thickness of ear skin between the vehicle-treated control group, DNCB-treated group, PA/DNCB-treated group, and COO/DNCB-treated group.

HE staining was used to determine the efficacy of aged COO in the treatment of mice with AD. As shown in Figure 4A, normal mouse skin histology was observed under a light microscope; the epidermal thickness and numbers of mast cells in the right ears were maintained at low levels, and no obvious lesions were observed. However, abnormal histological changes were observed in the dorsal skin and ears of DNCB-treated mice. Histopathology revealed that the ear tissues of mice in the DNCB-treated group were covered with squamous epithelium, with thickening of the epidermal layer, surface hyperkeratosis, hyperplasia of spindle cells, and dilation of blood vessels in the dermal layer, and the infiltration of inflammatory cells, such as lymphocytes and plasma cells, was observed. Compared with the vehicle-treated control group, the DNCB-treated group exhibited the dense infiltration of inflammatory cells. In addition, the mean thickness of the epidermal layer in the right ear in the DNCB-treated group was increased by 3.7-fold compared to the vehicle-treated control group (Figure 4B).

Mice treated with PA showed a significant improvement in inflammatory infiltration, amelioration of pathological changes, and improved therapeutic results, with the average thickness of the epidermal layer in the ear being 55% of that of the DNCB-treated group. The COO/DNCB-treated group showed similar results to those of the PA/DNCB-treated group after treatment. In the COO/DNCB-treated group, inflammatory cell infiltration was reduced in most areas, and pathological changes were ameliorated. However, focal vasodilatation and lymphocyte and plasma cell infiltration were still observed. The inflammatory cell infiltration was improved, and the epidermal layer was thinner compared to the DNCB-treated group. The epidermal thickening of the ear skin in the COO/DNCB-treated group was 48% of that in the DNCB-treated group.

### 3.4 Effect of COO on expression of cytokines in the ear

**FIGURE 5:**
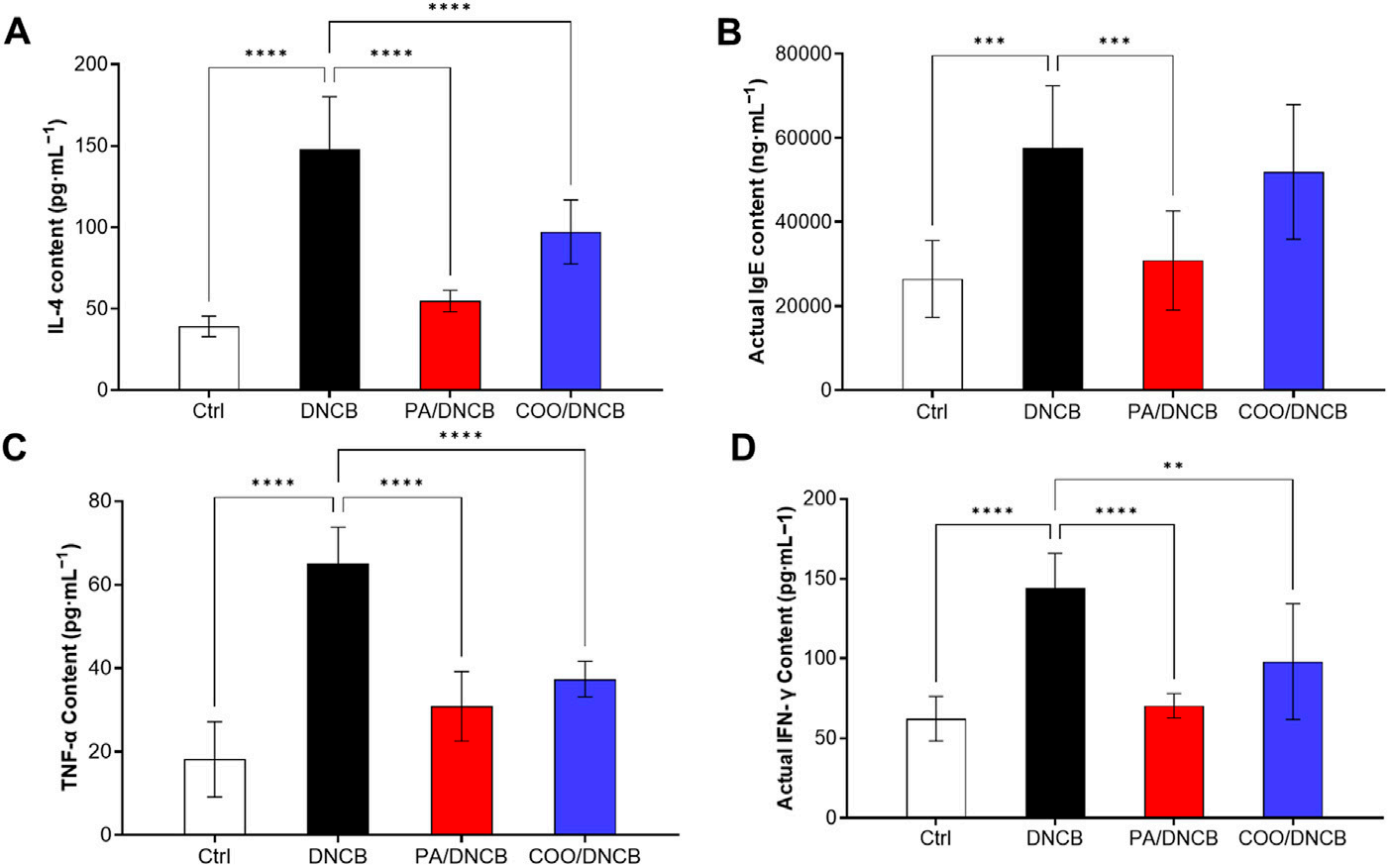
Effect of aged COO on serum levels of IFN-γ **(A)**, IL-4 **(B)**, IgE **(C)**, and TNF-α **(D)** in DNCB-induced mice.

As shown in Figure 5, repeated application of DNCB significantly increased the serum levels of inflammatory cytokines in mice in the DNCB-treated group compared with those in the vehicle-treated control group, as indicated by a 4.2-fold increase in IL-4 levels, a 3.4-fold increase in IgE levels, a 3.6-fold increase in TNF-α levels, and a 2.3-fold increase in IFN-γ levels. These results indicate that the dermatitis model has been successfully established.

The results showed that the IL-4 level in the COO/DNCB-treated group was 97.12 ± 19.62 pg·mL^−1^. Compared with the DNCB-treated group, the serum IL-4 level in the COO/DNCB-treated group was 40.6% lower than that of the DNCB-treated group but higher than that of the PA/DNCB-treated group, which had a serum IL-4 level of 54.67 ± 6.585 pg·mL^−1^ (Figure 5A).

IgE is an immunoglobulin and mediator of mast cell activation that plays a central role in type 1 hypersensitivity reactions and chronic allergic diseases (Wollenberg et al., 2021). When antigens cross-link with specifically bound IgE, mast cell degranulation is induced, followed by the secretion of histamine, leukotrienes, and chemokine-activated mediators, leading to itching and skin inflammation. The results showed that IgE levels were significantly reduced by 46.4% in the PA/DNCB-treated group compared to the DNCB-treated group (Figure 5B). The IgE level in the COO/DNCB-treated group decreased by about 9.9% compared with the DNCB-treated group, indicating that COO tended to reduce the inflammatory response in AD.

The analysis of TNF-α showed similar results. In response to inflammatory factors, TNF-α levels increase rapidly (Luo et al., 2022). The results showed that the level of TNF-α in the PA/DNCB-treated group was 30.86 ± 8.322 pg·mL^−1^, which was reduced by 52.5% compared with the DNCB-treated group (Figure 5C). The level of TNF-α in the COO/DNCB-treated group was 37.38 ± 4.281 pg·mL^−1^, which decreased by 42.5% compared with the DNCB-treated group.

IFN-γ is secreted mainly by activated T cells and is also known as an immune interferon (Fagundes et al., 2023). IFN-γ promotes the expression of MHC class II molecules, activates macrophages, and inhibits viral replication. The results showed that the level of IFN-γ in the PA/DNCB-treated group was 70.32 ± 7.597 pg·mL^−1^, which decreased by 51.2% (Figure 5D) compared with the DNCB-treated group. The IFN-γ level in the COO/DNCB-treated group was 97.94 ± 36.29 pg·mL^−1^, which decreased by 32.0% compared with the DNCB-treated group.

### 3.5 Metabolite analysis of COO

The compositions of COO were analyzed using Agilent MassHunter Unknowns Analysis software. As shown in Supplementary Table S1, forty-one metabolites were detected in COO, and the three major peaks (approximately 69.38%) were identified as oleic acid (112–80-1), *n*-hexadecanoic acid (57–10-3), and (*E*,*E*)-2,4-decadienal (CAS: 25,152–84-5). These results clearly indicate aged COO contains a considerable proportion of unsaturated fatty acids, among which oleic acid is the main metabolite (Zhang X.-L. et al., 2022). Supplementary Table S2 shows that the compositions of COO contain fatty acids (66.96%), esters (1.38%), alkanes (4.52%), aldehydes or ketones (25.67%). In addition, small amounts of nitrogen-containing metabolites (1.48%) were detected.

### 3.6 Molecular docking

The molecular docking results revealed that *n*-hexadecanoic acid, oleic acid, and octadecanoic acid had the good binding affinity, docking with filaggrin with the binding affinity at −6.979, −6.855, and −6.909 kcal·mol^−1^, respectively. All values are higher than that of the two eutectic ligands present in filaggrin 4pcw (−4.190 kcal·mol^−1^ and −4.144 kcal·mol^−1^, respectively). In general, the lower the resulting affinity is, the more stable the binding conformation is; in the absence of a positive control, binding affinities less than −6 kcal·mol^−1^ are generally considered to indicate more stable ligand-receptor interactions (Shityakov and Förster, 2014). Thus, the results confirm that the ability of the fatty acid ligands to bind to filaggrin is better than that of the eutectic ligands, indicating the good binding potential of each fatty acid to filaggrin.

**FIGURE 6:**
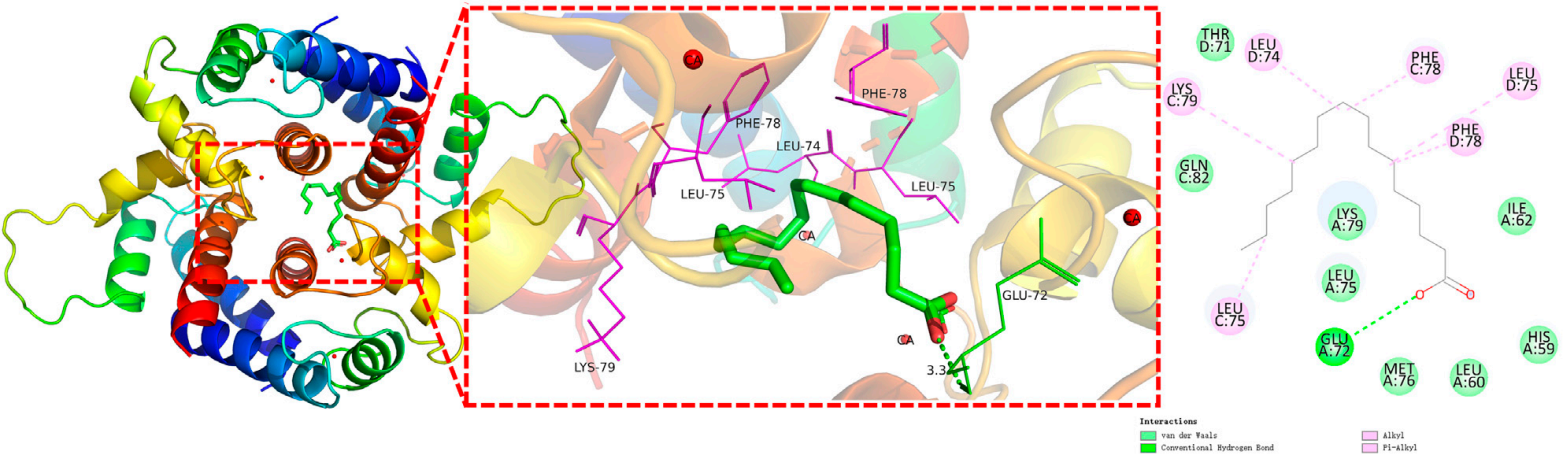
Interaction analysis of filaggrin with *n*-hexadecanoic acid docking results (π-bonds are shown as purple dashed lines; hydrogen bonds are shown as green dashed lines).

The surface binding pocket pattern diagram of *n*-hexadecanoic acid, which had the best effect (Figure 6), shows that the ligand bound within the pocket-like region inside the protein, and the two had good complementary shapes that were suitable for producing a specific binding effect. Figure 6 shows that the *n*-hexadecanoic acid ligand formed a hydrogen bond with the amino acid residue GLU A72 and six π/alkyl interactions or π-π stacking interactions with other amino acid residues (LEU C75, LYS C79, LEU D74, PHE C78, LEU D75, and PHE D78). We also observed that the binding sites of *n*-hexadecanoic acid and one of the proto-cocrystalline ligands were almost identical, which indicates that the two were well bound and confirms that the two had good affinity.

## 4 Discussion

In this study, we investigated the effect of aged COO on DNCB-induced AD-like skin damage in BALB/c mice. Typical AD symptoms such as erythema, papules, and severe itching could be clearly observed in the skin of mice after repeated alternating applications of DNCB. Analysis of the degree of ear skin thickness and weight swelling in the mice showed that the ears of the DNCB-treated group were more swollen than those of the vehicle-treated control group, and the thickness of the left and right ears was significantly different between the groups. When treated with PA or COO, the difference in thickness between the DNCB-treated and COO/DNCB-treated groups was significant compared with the vehicle-treated control group. The epidermis of ear specimens from the DNCB-treated group is hyperkeratotic and thickened, and the dermis is predominantly infiltrated by inflammatory cells and shows vasodilation, as shown by HE staining. We hypothesized that this thickening, in addition to hyperkeratosis, was associated with echinocyte proliferation and visible dermal vasodilation.

The monocyte/macrophage system that is stimulated by inflammation is activated by the release of many inflammatory factors, such as IL-6, IL-1β, TNF-α, and IFN-γ. To determine the changes of inflammatory markers in COO-treated AD mice, serum levels of IL-4, TNF-α, IgE, and TFN-γ were further analyzed. It is well known that IgE is an immunoglobulin and a mediator of mast cell activation that plays a central role in type 1 hypersensitivity reactions and chronic allergic diseases (Wollenberg et al., 2021). When antigen cross-links with specifically bound IgE, mast cell degranulation is induced, followed by secretion of histamine, leukotrienes, and chemokine-activating mediators, leading to itching and skin inflammation (Galli et al., 2008). As shown in this study, repeated treatment of the right ear of mice with DNCB resulted in infiltration of inflammatory cells into the dermis and increased serum levels of IgE. Topical application of COO reduced serum levels of IgE and significantly suppressed the number of inflammatory cells infiltrating the skin injury in AD mice.

Overexpression of IL-4 has been implicated as a key factor in inflammatory diseases such as AD (Elbe-Bürger et al., 2002). IL-4 induces the differentiation of initial CD4^+^ T cells into Th2 cells, which in turn produce more cytokines and initiate a type 2 inflammatory response (Cernescu et al., 2021). In addition, IL-4 and IL-13 together induce the conversion of immunoglobulins to IgE in B cells and stimulation of afferent neurons via the IL-4 receptor subunit-α (IL-4Rα) to promote pruritus (Bitton et al., 2020). In our results, serum levels of IL-4 were reduced in the COO/DNCB-treated group, suggesting that COO inhibits Th2 cell production by reducing serum levels of IL-4 and may play a bridging role in reducing serum levels of IgE.

TNF-α produced during the initiation phase of AD induces the production of various chemokines and adhesion molecules, leading to the recruitment and proliferation of inflammatory cells within the skin. In response to inflammatory factors, TNF-α levels increase rapidly (Luo et al., 2022). COO has been shown to have anti-inflammatory activity (Zhang et al., 2021; Zhou et al., 2024). Our experiments also showed that serum TNF-α levels were reduced in DNCB-induced BALB/c mice coated with COO, suggesting that the development of AD-like symptoms could be inhibited. COO has also previously been shown to inhibit TNF-α expression in activated human inflammatory cells (Zhang et al., 2021). Therefore, we hypothesized that the inhibitory effect of COO on inflammatory cell infiltration in the AD model may be mediated by blocking TNF-α expression and downstream chemokine/adhesion molecules.

In addition, IFN-γ is mainly secreted by activated T cells, also known as immune interferons (Fagundes et al., 2023). IFN-γ promotes the expression of MHC class II molecules, activates macrophages, and inhibits viral replication. In our experiments, serum levels of IFN-γ were reduced in mice coated with COO, suggesting that COO may have potential anti-AD effects.

Therefore, the experimental results showed that topical application of aged COO downregulated the expression of IgE, IL-4, IFN-γ, and TNF-α, and alleviated the development of AD-like symptoms in DNCB-induced BALB/c mice. Moreover, although aged COO had beneficial effects in ameliorating AD symptoms, they were weaker than those of prednisone acetate.

Based on the metabolite analysis of COO by GC/Q-TOF MS method and molecular docking analysis of three fatty acids with filaggrin binding capacity results, we conclude that fatty acids, which are the major chemical metabolites in COO, may have antipruritic and anti-inflammatory activities, and are potent active metabolites in the treatment of AD (Chang et al., 2020). In fact, many plant oils rich in polyunsaturated fatty acids play an important role as immunomodulators (Kildaci et al., 2021). These plant oils can be used to improve skin barrier function and reduce inflammation, and they are considered potential anti-wrinkle agents (Jung et al., 2007), but the saturation, concentration, and *cis-trans* structure of the double carbon bonds in these fatty acids of plant oils may influence the amelioration of AD symptoms (Venter et al., 2019; Olejnik et al., 2023). In addition, oral and topical oils for AD may have different mechanisms of action. Oral administration of COO may activate free fatty acid receptor 4 (FFA4) to increase regulatory T cells to ameliorate AD (Son et al., 2020). Topical application of aged COO may be the result of fatty acids in COO stably binding to the filaggrin of the epidermis, protecting the unique barrier structure of the cuticular surface layer, and preventing water loss, which may be the mechanism by which COO plays a role in the treatment of AD (Jung et al., 2007).

Filaggrin is not only a key component of epidermal keratin but also dissociates from keratin fiber bundles, transforming and degrading to form mixtures of water-absorbing amino acids that trap moisture in the skin through hydration (Cabanillas and Novak, 2016). Application of aged COO reduced transdermal water loss, increased skin hydration, alleviated DNCB-induced skin barrier alterations, and eliminated itchy scratching caused by dry skin. Meanwhile, aged COO inhibited matrix metalloproteinase (MMP)-1 activity and induced human type I procollagen synthesis (Zhang et al., 2024). Furthermore, in inflammatory skin, plant oils high in oleic acid or deficient or low in linoleic acid may cause additional structural damage to the stratum corneum, whereas plant oils rich in linoleic acid and saturated fatty acids may have beneficial effects (Poljšak and Kočevar Glavač, 2022). Therefore, we conclude that *n*-hexadecanoic acid, oleic acid, and octadecanoic acid have the best potential to be used as drug candidates for the treatment of AD, and filaggrin may be the drug target of the anti-AD effect (Earlia et al., 2019). These fatty acids play an important role in the anti-AD effect through coordinated action.

This study has some obvious limitations. During the experiment, the bare skin of the mice was wrapped in gauze or fed individually to prevent them from sucking on each other, but scratches on skin are still unavoidable. In addition, we only studied COO stored for 3 years, whether COO stored for longer periods has therapeutic effects needs to be determined. In addition, further cell experiments are needed to verify the effect.

## 5 Conclusion

In conclusion, administration of COO that was stored for 3 years attenuated the development of DNCB-induced AD symptoms in BALB/c mice and effectively ameliorated DNCB-induced AD symptoms. Infants and young children are susceptible to AD. However, due to the underdeveloped skin structure of infants and young children, the stratum corneum and epidermis of their skin are significantly thinner than those of adults, and the structure of the epidermis and dermis, as well as the skin appendages, are significantly different from those of adults, so the safety of the drug is preferred. Considering that COO has medicinal and edible values, we believe that aged COO is a promising anti-inflammatory and anti-allergic agent with significant efficacy, especially for infants and children. In the traditional use of aged COO, it is often believed that the longer aged COO is stored, the more effective it is. However, aged COO, especially oil that was stored for more than 5 years, tended to suffer from rancidity due to influences such as container, light, and storage time, which had significantly changed in the content of organic acids and advanced fatty acid esters. Not only did this oil have an unpleasant odor, but it could also affect the efficacy of treating AD. Therefore, our future focus is to investigate whether fresh COO is effective and how long COO should be stored to be effective, and to conduct further cell experiments to explore the mechanism.

## Data Availability

The original contributions presented in the study are included in the article/Supplementary Material, further inquiries can be directed to the corresponding authors.

## Abbreviations

AD, Atopic dermatitis; COO, *Camellia oleifera* Abel oil; DNCB, 2,4-dinitrochlorobenzene; ELISA, enzyme-linked immunosorbent assay; GC/Q–TOF MS, gas chromatography/quadrupole time-of-flight mass spectrometry; HE, hematoxylin-eosin; IFN-γ, interferon gamma; IL-4, interleukin 4; IgE, immunoglobulin E; TNF-α, tumor necrosis factor alpha.
