# Supplementary material for: Can aged Camellia oleifera Abel oil truly be used to treat atopic dermatitis?
Source: Front Pharmacol. 2024 Dec 4;15:1449994. doi: 10.3389/fphar.2024.1449994 (PMC11652156; doi:10.3389/fphar.2024.1449994)
Supplement: Supplementary file 1 [file DataSheet1.pdf]

Supplementary Material

Can aged *Camellia oleifera* oil truly be used to treat atopic dermatitis?

Xi-Lin Ouyang[1,*], Zhang-Lin Yuan[1], Xiao-Bing Chen[1], Hong-Wan Gan[2], Sen-Hui Guo[1], Juan Cai[1], Jing-Jing Zhong[2,*]

[1]Department of Pharmacy, Gannan Healthcare Vocational College, Ganzhou, China

[2]Department of Dermatology, Ganzhou People's Hospital, Ganzhou, China

*** Correspondence:**
Xi-Lin Ouyang
oyxl-xg@163.com
Jing-Jing Zhong
zhongjingjing2011@126.com

1 Supplementary Figures and Tables

Fig 1S TIC overlap plots of aged COO at 10:1 (blue) and 50:1 (green) split ratio, from which more peaks were detected with a split ratio at 10:1.

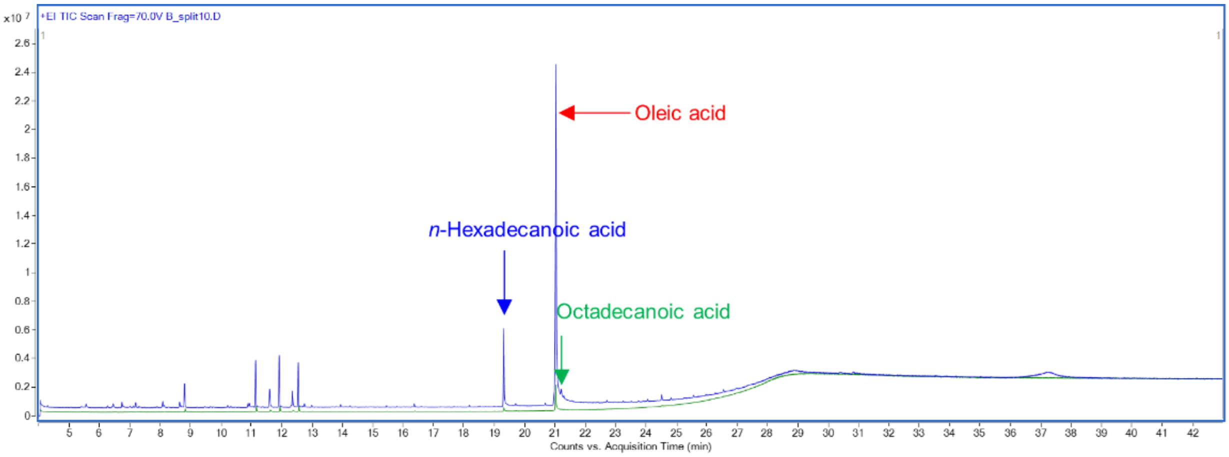

Table 1S Compositions of aged COO.

Retention time/min	Compound Name	Formula	CAS#	Aged COO
4.05	Hexane, 2,4-dimethyl-	C_8H_{18}	589-43-5	1.63
4.29	Hexane, 2,3,5-trimethyl-	C_9H_{20}	1069-53-0	0.22
5.42	2,3-Octanedione	$C_8H_{14}O_2$	585-25-1	0.15
5.56	Heptanal	$C_7H_{14}O$	111-71-7	0.80
6.26	Pentanal, 2,2-dimethyl-	$C_7H_{14}O$	14250-88-5	0.26
6.47	2-Heptenal, (*E*)-	$C_7H_{12}O$	18829-55-5	0.63
6.74	Hexanoic acid	$C_6H_{12}O_2$	142-62-1	0.98
6.97	4-Octanol	$C_8H_{18}O$	589-62-8	0.06
7.01	Furan, 2-pentyl-	$C_9H_{14}O$	3777-69-3	0.17
7.12	Decane	$C_{10}H_{22}$	124-18-5	0.18
7.19	Octanal	$C_8H_{16}O$	124-13-0	0.63
7.90	Cyclopentane, 1-ethyl-1-methyl-	C_8H_{16}	16747-50-5	0.19
8.08	2-Octenal, (*E*)-	$C_8H_{14}O$	2548-87-0	0.78
8.13	2,5-Hexanedione	$C_6H_{10}O_2$	110-13-4	0.36
8.27	Cyclopropane, pentyl-	C_8H_{16}	2511-91-3	0.16
8.64	Furan, 2-butyltetrahydro-	$C_8H_{16}O$	1004-29-1	0.66
8.80	Nonanal	$C_9H_{18}O$	124-19-6	2.34
9.19	Cyclopropane, pentyl-	C_8H_{16}	2511-91-3	0.06
9.66	2-Nonenal, (*E*)-	$C_9H_{16}O$	18829-56-6	0.29
10.22	Undecane	$C_{11}H_{24}$	1120-21-4	0.25
10.33	Decanal	$C_{10}H_{20}O$	112-31-2	0.51
10.95	2-Decenal, (*Z*)-	$C_{10}H_{18}O$	2497-25-8	0.13
11.07	Benzene, 1,3-bis(1,1-dimethylethyl)-	$C_{14}H_{22}$	1014-60-4	4.33

11.14	2-Decenal, (*E*)-	$C_{10}H_{18}O$	3913-81-3	2.52
11.92	2,4-Decadienal, (*E*,*E*)-	$C_{10}H_{16}O$	25152-84-5	5.04
12.55	2-Undecenal, (*Z*)-	$C_{11}H_{20}O$	53448-07-0	2.13
12.55	2-Undecenal, (*E*)-	$C_{11}H_{20}O$	2463-77-6	4.12
12.69	1H-Imidazole	$C_3H_4N_2$	288-32-4	0.11
12.75	1H-Pyrrole, 2,5-dihydro-	C_4H_7N	109-96-6	0.48
12.98	Hexadecane	$C_{16}H_{34}$	544-76-3	0.26
13.94	Ethanone, 1-(1,2,2,3-tetramethylcyclopentyl)-, (1R-cis)-	$C_{11}H_{20}O$	59642-07-8	0.36
14.26	Undecane	$C_{11}H_{24}$	1120-21-4	0.22
15.46	Decane, 2,6,8-trimethyl-	$C_{13}H_{28}$	62108-26-3	0.13
16.36	1-Heptadecene (Uncertain)	$C_{17}H_{34}$	/	0.38
19.31	*n*-Hexadecanoic acid	$C_{16}H_{32}O_2$	57-10-3	8.97
19.71	13-Octadecenal, (*Z*)-	$C_{18}H_{34}O$	58594-45-9	0.40
20.68	9-Octadecenoic acid (*Z*)-, methyl ester	$C_{19}H_{36}O_2$	112-62-9	0.33
21.02	Oleic Acid	$C_{18}H_{34}O_2$	112-80-1	55.37
21.20	Octadecanoic acid	$C_{18}H_{36}O_2$	57-11-4	1.64
24.51	trans-9-Octadecenoic acid, pentyl ester (Uncertain)	$C_{23}H_{44}O_2$	/	1.05
24.82	Heptacosane (Uncertain)	$C_{27}H_{56}$	/	0.33

Table 2S Composition analysis of different categories of aged COO.

	Fatty acid	Ester	Hydrocarbon	Aldehydes and Ketones	Other (alcohols or nitrogen)	Total
Aged COO	66.96%	1.38%	4.52%	25.67%	1.48%	100.01%
